# Symptomatic fever management among 3 different groups of pediatricians in Northern Lombardy (Italy): results of an explorative cross-sectional survey

**DOI:** 10.1186/1824-7288-39-51

**Published:** 2013-09-02

**Authors:** Alberto Bettinelli, Maria Cristina Provero, Felice Cogliati, Anna Villella, Maddalena Marinoni, Francesco Saettini, Mario Giovanni Bianchetti, Luigi Nespoli, Cino Galluzzo, Sebastiano Antonio Giovanni Lava

**Affiliations:** 1 Department of Pediatrics, San Leopoldo Mandic Hospital, Merate, Italy; 2 Department of Clinical and Experimental Medicine, Ospedale Pediatrico Filippo del Ponte, Varese, Italy; 3 Community Pediatrician, Merate, Italy; 4 Department of Pediatrics, San Gerardo Hospital, Monza, Italy; 5 Department of Pediatrics, San Giovanni Hospital, Bellinzona and University of Bern, Bern, Switzerland; 6 Department of Pediatrics, Fatebenefratelli Hospital, Erba, Como, Italy

**Keywords:** Fever, Pediatrician attitudes, Acetaminophen (paracetamol), Ibuprofen

## Abstract

**Background:**

In the care of feverish children, symptomatic management is pivotal. Thus, the Italian Pediatric Society has recently published guidelines on fever management in children. Our aim was to investigate whether pediatric hospitalists, community pediatricians and pediatric residents differ in their every-day clinical practice with respect to symptomatic management of feverish children.

**Methods:**

79 out of 118 physicians involved in pediatric care in an area of Northern Lombardy (Italy) filled in a modified version of the questionnaire derived from the Swiss national survey on symptomatic fever management.

**Results:**

Pediatric hospitalists (N = 29), community pediatricians (N = 30) and pediatric residents (N = 20) did not differ with respect to temperature threshold for symptomatic fever treatment, role of general appearance in modulating the threshold for fever management, first choice antipyretic drug, frequency of ibuprofen prescription, prescription of physical antipyresis, influence of exaggerated fear of fever on its management and potential to reassure families about this fear.

On the other side, some significant differences were found. Pediatric residents more frequently lower the treatment threshold in children with a past history of febrile seizures (P < 0.001) and prescribe an aggressive treatment for fever not responding to the first antipyretic drug (P < 0.01) than their more experienced colleagues. Community pediatricians represent the unique investigated group using homeopathic remedies, both in the acute setting (P < 0.001) as well as a prophylaxis (P < 0.0001). Finally, paediatric residents less often (P < 0.05) stated to encounter exaggerated fear of fever among parents than their more experienced colleagues.

**Conclusions:**

The present explorative inquiry globally shows limited discordance among pediatric residents, community pediatricians and pediatric hospitalists with respect to symptomatic fever management.

## Background

Since symptomatic management of fever is crucial both in self-limiting (mostly viral) and in severe (mostly bacterial) febrile illnesses [1,2], the Italian Pediatric Society has recently published guidelines on fever management in children [3,4].

Interestingly, some differences in diagnostic and therapeutic patterns among pediatric hospitalists, community pediatricians and paediatric residents have been observed [5,6]. Our aim was to investigate whether pediatric hospitalists, community pediatricians and pediatric residents differ in their every-day clinical practice with respect to the adherence to available guidelines on fever management.

## Methods

Between June and September 2012, we invited some of the physicians involved in pediatric care in the Provinces of Lecco, Como and Varese (Northern Lombardy, Italy) to fill in a questionnaire dealing with symptomatic management of fever. For this purpose, we slightly modified the close-ended questionnaire developed for the Swiss national survey on symptomatic fever management [7,8]. The 118 invited physicians included 29 pediatric residents, 48 community pediatricians and 41 pediatric hospitalists. While pediatric hospitalists worked at 4 different hospitals, pediatric residents all worked at the same institution.

To identify potential differences among the 3 groups of physicians, we analyzed the answers to 12 written questions that elicit information about the following: (1) rectal temperature threshold for initiating pharmacologic management of fever in a 3-year-old child who appears comfortable (possible answers: <38.0°C, 38.0-38.4°C, 38.5–38.9°C, 39.0–39.4°C, or ≥39.5°C); (2) the importance of a child’s general appearance in choosing the temperature threshold for initiating pharmacologic treatment of fever (never or rarely, sometimes, or often important); (3) the value of a child’s history of febrile seizures in choosing the temperature threshold for initiating pharmacologic treatment of fever (never or rarely, sometimes, or often important); (4) the prescribing of acetaminophen (paracetamol) as the first choice drug in the management of fever (first choice or not first choice); (5) the prescribing of oral ibuprofen for fever (never or rarely, sometimes, or often prescribed); (6) the management of a comfortable child with fever that is nonresponsive to an antipyretic drug (wait and see, replace the initial drug with a new one, or add a second drug to the first one); (7) the prescribing of physical methods of antipyresis (never or rarely, sometimes, or often prescribed); (8) the prescription of homeopathic remedies for the acute management of fever (yes or no) or for (9) its prevention (yes or no); (10) the perceived frequency of an exaggerated fear of fever among parents (never or rarely, sometimes, or often present); (11) the influence of exaggerated fear of fever on the drug management of fever (never or rarely, sometimes, or often lower threshold because of parental worries); and (12) the possibility of educating families about the fear of fever (never or rarely, sometimes, or often possible).

Ordered categorical responses to the questionnaire were assigned a numerical score. Numerical data were analyzed using the Kruskal–Wallis test and the Bonferroni-Dunn post hoc procedure. The Fisher exact test was used to analyze proportions. Significance was assigned at P < 0.05 (two-tailed).

## Results

**Figure 1:**
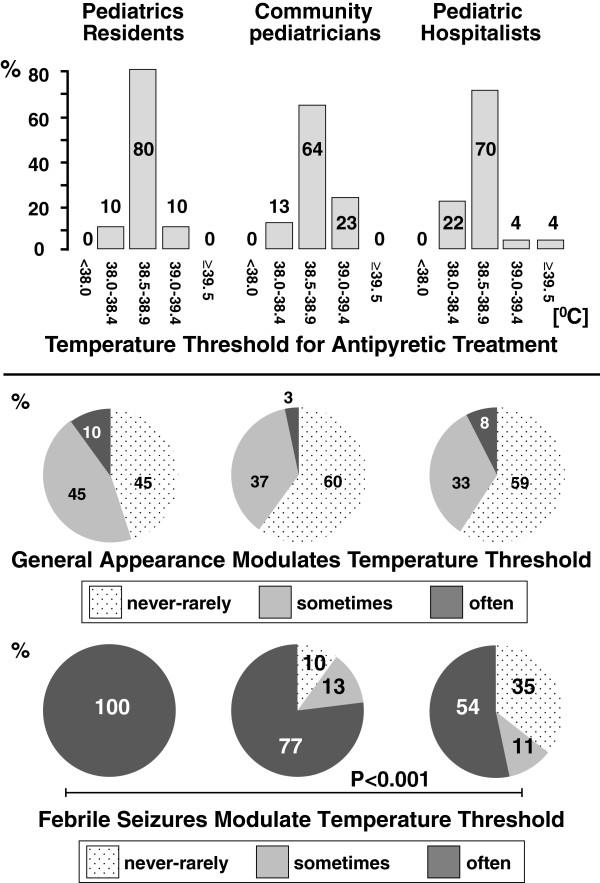
**Initial symptomatic management of fever.** The upper panel depicts the rectal temperature threshold for initiating symptomatic drug treatment in a 3-year-old child, who is nontoxic in appearance. The pie charts depict the role of the child’s general appearance (middle panel) and that of a past history of febrile seizures (lower panel) in modulating the temperature threshold to initiate symptomatic fever treatment. When statistical significance was reached, a horizontal bar indicates the degree of significance.

Seventy-nine (67%) out of the 118 invited physicians answered the questionnaire (Table 1). The rectal temperature threshold for symptomatic fever treatment was similar in the three study groups [Figure 1, upper panel]. Furthermore, ≥45% of the participants never or rarely lower the treatment threshold in front of a febrile child who is presenting with a reduced general appearance, without any difference between the 3 groups [Figure 1, middle panel]. Finally, in all groups ≥54% of the participants often reduce the temperature threshold for initiating an antipyretic treatment in children with a past history of febrile seizures [Figure 1, lower panel]. This attitude is more frequent (P < 0.001) among pediatric residents (100%) than among pediatric hospitalists (54%), without any significant difference between community pediatricians (77%) and the other two groups.

**Table 1:** Number of invited and respondent participants and their gender in the 3 groups of pediatricians (M : F ratio = male-to-female ratio)

	**Total number of participants**	**Pediatric residents**	**Community pediatricians**	**Pediatric hospitalists**
Respondents/Invited (percentage of respondents)	79/118 (67%)	20/29 (69%)	30/48 (60%)	29/41 (71%)
Respondents, M : F ratio	16 : 63	3 : 17	7 : 23	6 : 23

**Figure 2:**
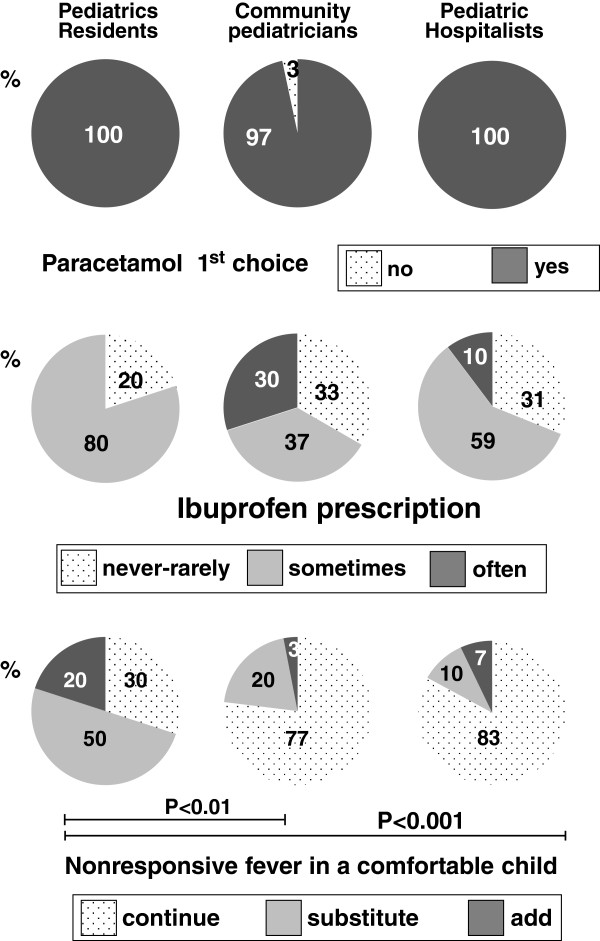
**Treatment regimen.** The upper panel depicts percentage of participants for whom acetaminophen (paracetamol) remains the first choice antipyretic drug. The pie charts in the middle panel illustrate the frequency of prescription of the oral non-steroidal anti-inflammatory agent ibuprofen. The lower panel depicts the management of a comfortable child whose fever is nonresponsive to the first antipyretic drug. When statistical significance was reached, a horizontal bar indicates the degree of significance.

In all groups, ≥97% of the participants prescribe acetaminophen as the first choice antipyretic drug (no significant difference was noted between the 3 groups) [Figure 2, upper panel]. Ibuprofen is sometimes or often used by ≥67% of physicians in each of the investigated categories, with 33% or less using it only rarely [Figure 2, middle panel]. No significant differences were found among the 3 groups. The management of a comfortable child whose fever does not respond to the first antipyretic drug differs among groups: pediatric residents replace the first drug with another antipyretic (50%) or, more rarely, add a second drug to the existing regimen (20%) more frequently than community pediatricians (20% and 3%, respectively; P < 0.01) and pediatric hospitalists (10% and 7%, respectively; P < 0.001) [Figure 2, lower panel].

**Figure 3:**
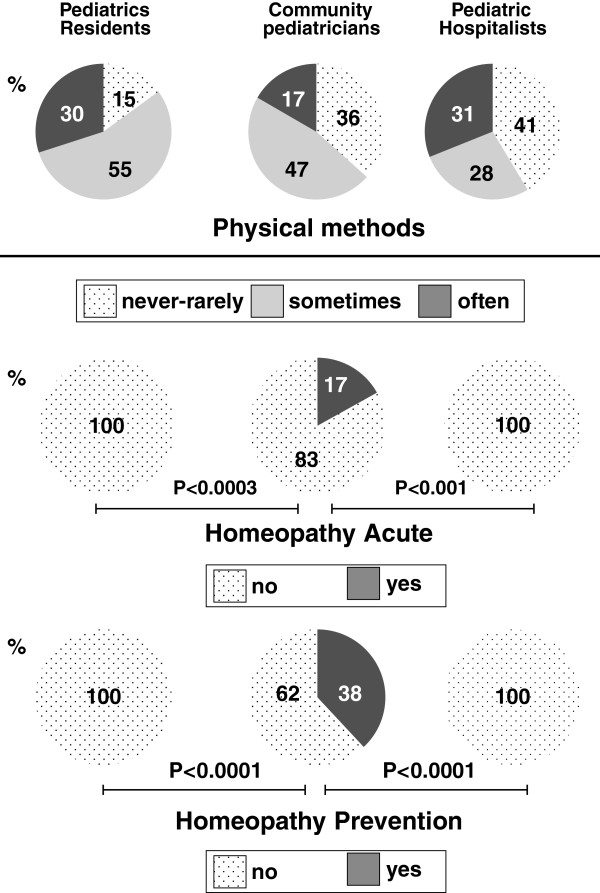
**Physical antipyresis and homeopathy.** The upper panel depicts the role of physical antipyresis in the acute management of a feverish child. Furthermore, the role of homeopathy in the acute management (middle panel) and in the prevention of fever (lower panel) is shown. When statistical significance was reached, a horizontal bar indicates the degree of significance.

In all groups, physical methods of antipyresis are used at least sometimes by ≥59% of the participants, without significant differences between groups [Figure 3, upper panel]. Hospitalists and residents never prescribe homeopathic remedies [Figure 3, middle and lower panel]. On the contrary, community pediatricians sometimes prescribe homeopathy both in the acute setting (17%; P < 0.001) as well as prophylaxis (38%; P < 0.0001).

**Figure 4:**
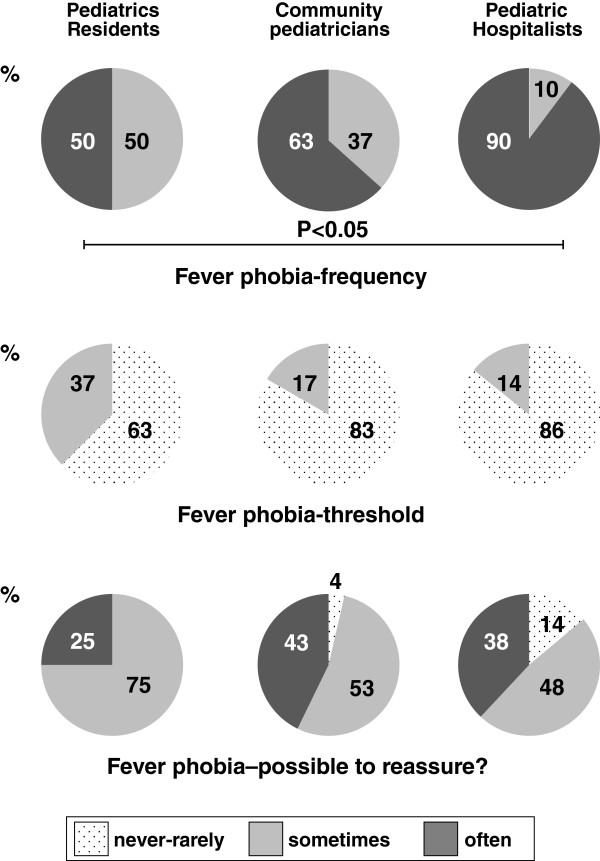
**Fever phobia.** The upper panel depicts the frequency of exaggerated fear of fever among parents, as perceived by the participants. The middle panel shows the influence of exaggerated fear of fever on the temperature threshold to start a symptomatic treatment of fever, while the lower panel denotes the potential to educate and reassure families about the fear of fever, as perceived by the participants in their everyday clinical practice. When statistical significance was reached, a horizontal bar indicates the degree of significance.

Participants from all the 3 groups consider that exaggerated fear of fever is frequent among parents. Nevertheless, the stated occurrence [Figure 4, upper panel] is lower (P < 0.05) among pediatric residents (50% of the participants state that fear of fever is frequent) than among community pediatricians (63%) and pediatric hospitalists (90%), with no significant difference between the latter two groups. In all groups ≥63% of the participants state that they rarely or never lower the temperature threshold [Figure 4, middle panel] for initiating a treatment in order to calm worried parents (without significant difference between the 3 groups). Similarly, in all groups, ≥86% of the participants consider that it is sometimes or often possible to educate and reassure families [Figure 4, lower panel] about the fear of fever (without significant differences between the 3 groups).

## Discussion

The present explorative inquiry globally shows limited discordance among the 3 groups of investigated pediatricians, with only 5 issues reaching a statistically significant difference, as depicted in Table 2.

**Table 2:** Distinctivenesses in symptomatic fever management among pediatric residents, community pediatricians and pediatric hospitalists practising in an area of Northern Lombardy

	**Pediatric residents**	**Community pediatricians**	**Pediatric hospitalists**
Febrile seizures modulate temperature threshold	**++**	**+**	**+**
Aggressive treatment of nonresponsive fever in a comfortable child	**+**	**-**	**-**
Homeopathy in the acute setting	**-**	**+**	**-**
Homeopathy as prophylaxis	**-**	**+**	**-**
Exaggerated fear of fever frequent	**+**	**++**	**++**

The symbol ++ means very common, the symbol + frequent and the symbol – rare.

First, the rectal temperature threshold for symptomatic fever treatment was similar (38.5°-39.0°) to that reported in a recent Italian study performed among pediatricians [9]. Second, it is currently advised that antipyretic drugs should be prescribed only when fever is associated with evident discomfort [3,4,10-13]. The present survey indicates that the child’s general appearance only rarely modulates the threshold for symptomatic fever treatment throughout the analyzed groups of pediatricians. Third, antipyretics are not effective in preventing febrile seizures and should therefore be avoided [3,4,10-15]. According to our results, more experienced hospital-based pediatricians less frequently differ from this recommendation than their younger colleagues.

Fourth, in all interviewed groups, ≥97% of the participants stated to prescribe acetaminophen as the first choice antipyretic. The fact that ibuprofen is sometimes or often used by ≥67% of participants indicates that this non-steroidal agent is often used as an alternative to acetaminophen. Both results reflect the attitudes of Italian Pediatricians reported in a recently published survey [9]. Fever not responding to a first antipyretic agent does not signalize the presence of a serious or dangerous illness [16]. However, high temperature that does not go down may be associated with a suffering and uncomfortable child and should therefore be effectively managed [3,4,7,8,10-12]. Therefore, in our survey we explicitly asked about the management of a nonresponsive fever in a comfortable child. As compared to pediatric hospitalists and community pediatricians, pediatric residents more often aggressively treat a comfortable child whose fever is not going down, either by replacing the first antipyretic drug or by adding a second agent. This attitude likely reflects greater worries about this condition.

Sixth, physical methods of antipyresis [17,18] are used at least sometimes by ≥59% of the respondents throughout the analyzed groups. This roughly mirrors the results of a recent study performed in a larger sample of Italian Pediatricians [9], but does unfortunately not recflect the guideline recommendations [3,4,7,8,11,13,15]. Seventh, homeopathy is a controversial practice founded by the German physician Samuel Hahnemann in the late 18^th^ century [19]. In our sample, homeopathic remedies are prescribed exclusively by a minority of community pediatricians. This might reflect an attempt to reassure patients and caregivers by prescribing innocuous remedies with no proven effect beyond placebo.

The presence of several unrealistic fears about fever, firstly noted in 1980, has been called ‘fever phobia’ [20]. Since then, several studies have recognized its presence both among caregivers as well as health professionals [20-25]. Intriguingly, pediatric residents appear to encounter fever phobia less often than their more experienced hospital-based colleagues. This is surprising, since residents more often declared to use non-evidence based practices such as a more aggressive treatment of a nonresponsive fever or the “prophylactic” prescription of antipyretics for children with a history of febrile seizures (Table 2). Since the spectrum of patients cared for by residents, hospitalists and community-pediatricians is likely identical, it is tempting to assume that young residents tend to underrecognize fever phobia. Part of the reason for this tendency might reside in the fact that physicians themselves (and, we guess, residents maybe stronger than more experienced clinicians) can be victims of fever phobia [22,26,27].

Our results must be interpreted with an understanding of some methodological limitations. First, since a study based on a small number of participants has little chance of producing clear-cut conclusions, the results of our explorative survey with 3 small groups of residents, hospitalists and community-based pediatricians might deserve confirmation with a larger sample of participants. Second, the results of this study, performed in Northern Lombardy, cannot be automatically generalized to other regions of Italy or to other Countries. In fact, data comparing the mentioned 3 groups of physicians in other Countries are currently not yet available.

Third, although self-reported physicians’ questionnaires have been frequently used, answers on surveys that ask doctors how they deal with specific conditions, sometimes differ from their everyday clinical practice [8]. Finally, the provided data are simply quantitative. The present survey did not investigate the reasons underlying the answers provided by the interviewed physicians. Thus, all the explanations are speculative. In order to analyze possible reasons explaining the differences in symptomatic fever management among the 3 study groups, a qualitative study based on in-depth interviews would be helpful.

In conclusion, this explorative study demonstrates limited discordance among pediatric residents, community pediatricians and pediatric hospitalists with respect to symptomatic fever management (Table 2). Larger confirmatory studies deserve to be performed.

## Competing interests

The authors declare that they have no competing interests.

## Authors’ contributions

AB and SAGL designed the study, performed statistical analysis and wrote the initial draft. SAGL and MGB prepared the figures. MCP, FC, AV, MM, FS, LN and CN took the verbal consent, administered and collected the questionnaires. All authors read and approved the final manuscript.
